# Home treatment as an add-on to family-based treatment for adolescents with anorexia nervosa compared with standard family-based treatment and home-based stress reduction training: study protocol for a randomized clinical trial

**DOI:** 10.1186/s40337-023-00861-5

**Published:** 2023-08-14

**Authors:** Nicole Besse-Flütsch, Claudia Bühlmann, Natalie Fabijani, Gian Giacomo Ruschetti, Lukasz Smigielski, Dagmar Pauli

**Affiliations:** Department of Child and Adolescent Psychiatry, Psychiatric University Hospital Zürich, University of Zürich, Neumünsterallee 3, 8032 Zurich, Switzerland

**Keywords:** Eating disorders, Home treatment, Adolescents, Family-based treatment, Anorexia nervosa, Randomized clinical trial, Mindfulness-based stress reduction training

## Abstract

**Background:**

Family-based treatment (FBT) is currently the most effective evidence-based treatment approach for adolescents with anorexia nervosa (AN). Home treatment (HT) as an add-on to FBT (FBT-HT) has been shown to be acceptable, feasible and effective. The described three-arm randomized clinical trial (RCT) is intended to investigate whether FBT-HT demonstrates higher efficacy compared to standard outpatient FBT with supplemental mindfulness-based stress reduction training (FBT-MBSR).

**Methods:**

This RCT compares FBT-HT to standard outpatient FBT and FBT-MBSR as a credible home-based control group in terms of efficacy and delivery. Adolescents with AN or atypical AN disorder (*n* = 90) and their parent(s)/caregiver(s) are to be randomly assigned to either FBT, FBT-HT or FBT-MBSR groups. Eating disorder diagnosis and symptomatology are to be assessed by eating disorder professionals using standardized questionnaires and diagnostic instruments (Eating Disorder Examination, Eating Disorder Inventory, Body Mass Index). In addition, parents and caregivers independently provide information on eating behavior, intrafamily communication, stress experience and weight. The therapeutic process of the three treatments is to be measured and assessed among both participants and care providers. The feasibility, acceptability and appropriateness can thus also be evaluated.

**Discussion:**

We hypothesize that FBT-HT will be an acceptable, appropriate and feasible intervention and, importantly, will outperform both established FBT and FBT-MBSR in improving adolescent weight and negative eating habits. Secondary outcome measures include the reduction in the stress experienced by caregivers, as well as the regulation of perceived expressed emotions within the family, while the intrafamily relationships are hypothesized to mediate/moderate the effectiveness of FBT. The proposed study has the potential to enhance the scientific and clinical understanding of the efficacy of FBT for AN, including whether the addition of HT to FBT versus another home-based adjunct intervention improves treatment outcomes. Furthermore, the study aligns with public health priorities to optimize the outcomes of evidence-based treatments and integrate the community setting.

*Trial registration* This study is registered at ClinicalTrials.gov (NCT05418075).

## Introduction

Anorexia nervosa (AN) is a severe disorder with high morbidity, chronicity and mortality rates [15] associated with substantial suffering on personal, familial and social levels [33, 35]. Recent systematic reviews indicate that parental involvement through family-based treatment (FBT), especially in younger adolescents (12–15 years), is currently the most evidence-based treatment approach for adolescents with AN. [8, 16, 20, 38]. FBT is a well-established treatment modality that aims to normalize adolescents’ weight and eating behaviors [14] by empowering parents as supportive caregivers [11]. Notably, approximately 40–50% of adolescents participating in FBT show complete and stable remission in long-term follow-up studies, meaning that a substantial proportion of patients is still not making sufficient therapeutic progress [25]. Moreover, families with strong parental emotionality show even worse outcomes with FBT [1]. Accordingly, a better understanding of the moderators and mediators of FBT's treatment effects is needed to determine what additional therapies should potentially be offered when standard treatment is not sufficient [20]. In addition, FBT is generally seldom integrated into clinical practice and therapeutic settings [28]. Family and work commitments of parents are reported as common reasons for therapists’ reluctancy to involve parents in treatment [19].

Outreach services generally offer greater flexibility in terms of time and location, enabling the support of family structures in their ordinary environment, which minimizes the burden on all family members [6]. Such services are generally used to intensify treatment during a severe course of illness [24] and, in treatment for eating disorders, to prevent inpatient hospital admissions [30]. Transferring treatment strategies to the home setting is the key challenge to achieving sustained symptom reversal and long-term success in childhood and adolescent mental health treatment [36]. Specifically for adolescents with eating disorders, incorporating home treatment (HT) has been shown to be feasible and acceptable [9, 17], with a significant increase in treatment effects [30]. FBT is particularly well suited as a supplement to HT because the specific eating situations observed in FBT as possible maintaining factors [10] require effective management in the home setting. A recent qualitative study examined parents’ experiences and perceived stress in the context of FBT treatment and symptom persistence in their child [37]. HT could be a supportive element to reassure parents in their tasks prescribed in the FBT sessions and help the family to (re-)establish more beneficial and supportive interactions.

As postulated by Omer and London [29], the therapeutic relationship is the most important factor for therapeutic success. Although several HT programs for adolescents with psychiatric disorders have been developed and evaluated as effective [2, 24], to date, no studies have been able to demonstrate that the greater effectiveness of home-based care was not simply owing to the more intense therapeutic relationship, which may result from the additional time spent with the patient or the greater intimacy of the therapy environment.

The purpose of the proposed study (randomized clinical trial) is to compare the three outpatient treatments (FBT, FBT-HT, FBT-MBSR) for adolescents with AN. We hypothesize that FBT-HT is acceptable, appropriate and feasible and, importantly, outperforms both established FBT alone and FBT supplemented with mindfulness-based stress reduction training (FBT-MBSR) in terms of adolescent weight gain and eating disorder psychopathology. Our primary endpoint is remission of AN symptoms, defined as an increase in body weight as measured by BMI, and a decrease in eating disorder pathology as measured by the Eating Disorder Examination (EDE) total score and EDI-2 total score. Secondary endpoints include stabilization of somatic health status, as measured by vital signs, as well as an improvement in psychosocial well-being and reduction in perceived stress. We hypothesize that FBT-HT will lead to stress reduction in caregivers as well as improvement in interfamily relationships and regulation of perceived and expressed emotions within the family. In addition, we expect that balanced and relaxed family dynamics (integrated in further exploratory analyses as a mediator/moderator variable) will lead to increased effectiveness of FBT and thus improved patient-related outcomes.

## Methods

### Participants

Participants are to include a consecutive sample of 90 adolescents (aged 12–18 years) with full-syndrome AN or atypical AN (as well as their caregivers) that have been referred to the outpatient eating disorders unit at the Department of Child and Adolescent Psychiatry of the University Psychiatric Hospital, Zurich, Switzerland. Only patients with atypical AN who have all psychopathological symptoms of AN but whose percent mean body mass index (BMI) is clinically unremarkable according to the Centers for Disease Control and Prevention (CDC) growth charts [23] will be included in this study. Participants will be randomly assigned to one of three treatments (FBT, FBT-HT, FBT-MBSR). The inclusion criteria are as follows: (1) living with at least one adult caregiver,(2) willingness and ability to engage in family therapy; (3) being medically stable for outpatient treatment, as determined by a physician; (4) lack of comorbidities that contraindicate psychotherapy (e.g., psychosis); (5) an IQ greater than 75 (as determined by testing/clinical impression by a professional); (6) adequate German language skills; (7) residency within the Canton of Zurich, as outreach services necessary for two of the treatment groups are limited to this geographical region.

### Procedures

**Fig. 1:**
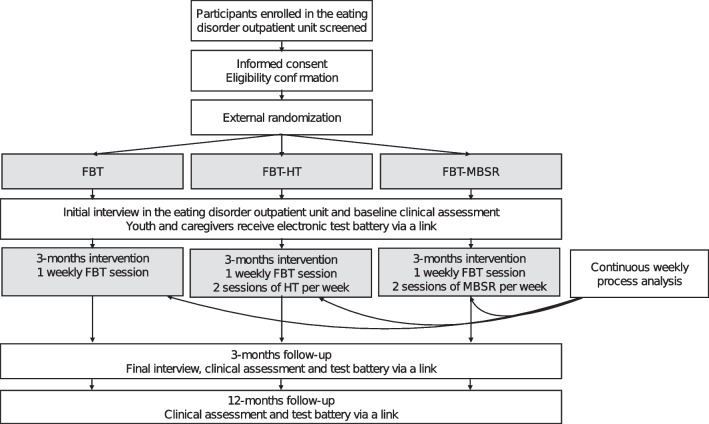
Schedule of study procedures. Abbreviations: *FBT* family-based treatment, *FBT-HT* home treatment as an add-on to FBT, *FBT-MBSR* home treatment as an add-on to a mindfulness-based stress reduction program, *HT* home treatment

At the enrollment phase, families will be screened for inclusion criteria in a telephone interview (see Fig. 1). If the study requirements are met, the adolescents and their caregivers will be informed about the study procedures during an initial contact meeting and will receive the consent form. External randomization will be performed by a researcher who is not involved in the study. All patients will undergo an initial clinical assessment to determine clinical parameters (e.g., weight, height, BMI, pulse). The therapists will discuss the initial FBT-interventions with the family and, after randomization, inform the family whether they will additionally receive guidance for FBT-HT or FBT-MBSR. After the initial interview, both the adolescents and their parents will receive a web-based link to an electronic test battery assessing psychopathology and interfamily relationships to be completed within 7 days. The study intervention will last 3 months. At the end of the treatment phase, the clinical staff will again collect clinical parameters, and another link to an electronic test battery will be sent. During the 3-month treatment phase, weight, blood pressure, and pulse will be recorded weekly. In addition, treatment progress is to be assessed by both patients and therapists after each FBT consultation. After 12 months, families will be contacted by email and asked to complete the final test battery.

### Interventions

Professionals providing FBT in the three treatment groups will follow the FBT manual [26] and will be trained during a 2-day introductory course in FBT for AN offered by the Training Institute for Child and Adolescent Eating Disorders and led by Daniel Le Grange and James Lock. All study participants will receive a weekly FBT session with a trained FBT clinician. In addition, the FBT-HT group will receive two 60–90-min consultations per week at home, conducted by a qualified FBT-HT professional. The FBT-MBSR group will receive two 60–90-min consultations delivered by a qualified MBSR trainer. FBT-HT professionals are to be graduate nurses or social workers with graduate professional degrees and experience in inpatient and outpatient treatment of adolescent eating disorders. The concept of milieu therapy appears against the background of an interdisciplinary understanding as a holistic treatment approach for children and adolescents with psychiatric disorders [18]. Milieu therapy is delivered by nurses and/or social workers and therefore requires fewer psychotherapeutic resources and is less costly. The relational style of milieu therapists differs from that of psychotherapists, it is predominantly daily, very action-oriented and less detached. The MBSR trainers are to be psychologists with a master’s degree who have completed a one-semester workshop on MBSR at the University of Zurich. FBT-HT professionals are to be trained in FBT treatment of adolescents with eating disorders through a workshop and ongoing group supervision by a clinical psychologist with a master’s degree and specific experience with AN. All professionals providing FBT will receive weekly supervision by a psychologist holding a master’s degree or a doctoral degree as part of the standard organizational procedure.

#### Family-based treatment

FBT is a manualized three-phase intensive outpatient treatment, in which parents play an active and positive role in helping their child (1) reach the weight expected for their age and height, (2) regain control over their eating and (3) promote the child’s normal development through in-depth discussion of the critical developmental issues affecting their child. FBT will be conducted by state-certified psychotherapists in weekly sessions of 60 min together with the patient and the caregiver(s) in the facilities of the Clinic for Child and Adolescent Psychiatry. Siblings are not actively involved in treatment and will not be included in the evaluation. In the first phase, parents/caregivers are empowered to be responsible for the adolescent’s eating behaviors and weight by providing meal structure in addition to preparing and serving meals for the adolescent. Once weight is restored and a more conflict-free eating situation is achieved, phase two follows, in which control over eating is returned to the adolescent. Phase three aims to prevent relapse, but also to reintegrate the adolescent into a daily routine appropriate for their developmental age.

#### FBT: home treatment

FBT-HT is an adapted intervention that complements manualized outpatient FBT with HT. During the treatment period of 12 weeks, the HT professional is to visit the family twice a week for 60–90 min at home or, at the family’s request, another agreed upon location. The HT intervention starts with a diagnostic component undertaken to assess eating-disorder maintaining factors of the individual (e.g., body-checking, frequent self-weighing) and of family interactions (e.g., parental criticism, unhelpful family communication around meal-times). Subsequently, the individual HT goals are set by involving the patient and parent(s)/caregiver(s), as well as the FBT-therapist that collaborates with the HT professional. The intervention is then carried out through regular visits, during which the HT professional takes part in family meals. The HT professional addresses crucial issues regarding interactions between the patient and the parents and gives advice on how to overcome them. The main goal is to provide practical support to parents in refeeding their adolescent, the key task of the first phase of FBT. Another important part of the intervention is to foster the patients’ and families’ resilience factors and promote the patient’s resources in everyday social life (e.g., restart hobbies, meet friends). HT sessions require the participation of at least one parent/caregiver and can be administered in different settings involving the patient, siblings or other significant people in the family environment.

#### FBT: MBSR (control group)

During the 12-week treatment period, FBT-MBSR is to include two 30-min consultations per week to practice and implement mindfulness for patients. The MBSR interventions are manualized exercises and meditations based on the 8-week MBSR program described by Kabat-Zinn [21]. The exercises and meditations are adapted to the needs of adolescents and supplemented with practices from mindfulness exercises with children and adolescents in psychotherapy [13]. To not to deviate further from the 8-week program manual, MBSR sessions are not timed to coincide with HT sessions. To support standardization and daily practice, meditation podcasts are provided, each lasting between 6 and 13 min. The MBSR therapist makes home visits for sessions or offers online appointments upon request. Each session includes meditation, a mindfulness exercise and also an opportunity to share and receive answers to possible questions.

### Measures

Demographic data as well as previous or current psychiatric treatment data will be collected from patients/their parent(s)/caregiver(s). During registration by telephone, patients are to be asked about the required language skills. All measurement instruments (see Table 1) are already digitized and to be electronically transmitted for the study via REDCap, a secure web-based electronic data collection application. The digitized version of the Eating Disorder Examination (EDE) [7] was previously investigated for equivalence with the original face-to-face EDE interview by our group; this study indicated that both instrument versions are overall equivalent in their measured scales, additionally showing that, as a diagnostic tool, the online version is generally answered more honestly [3].

**Table 1:** Summary of measures

Construct	Measure	Respondent/format	Time point		
			Week 0	Week 12	12 months
Descriptives	Age, gender, comorbid diagnosis, menstrual status, driven sporting activities, premorbid BMI, medical history	A, C	×		
Primary outcomes	EDE	A_os_	×	×	×
	EDI-2	A_os_	×	×	×
	BMI	A	×	×	×
Secondary outcomes					
Health-related quality of life	KID SCREEN-27	A_os_	×	×	×
Vital parameters	Blood pressure and pulse	A	×	×	×
Expressed emotion	BDSEE	A_os_	×	×	×
Stress perception	PSS-10	C_os_	×	×	×
Mediator/moderator					
Family functions	FB-K	A_o_, C_o_	×	×	×
Exploratory outcome					
Child behavior	CBCL	C_oa_	×	×	×
Implementation characteristics					
Therapy evaluation	FBB	A_o_, C_o_		×	
Process analysis of methods	BPT	A, C, T	Weekly		

*A* adolescent, *C* caregiver, *T* therapist, *o* online, *os* online self-reporting, *oa* caregiver online reporting on adolescent, *BDSEE* Brief Dyadic Scale of Expressed Emotion, *BMI* body mass index, *BPT* Berner Patienten- Therapeutenstundenbogen, *CBCL* child behavior checklist, *ED* eating disorder, *EDE* eating disorder examination, *EDI-2* eating disorder inventory, *FB-K* family questionnaire, *FBB* treatment satisfaction (in German: Fragebogen zur Beurteilung der Behandlung), *PSS* Perceived Stress Scale

#### Implementation

The feasibility and acceptance of FBT-HT have already been demonstrated in our preceding pilot project [9], which also indicated the rates at which families eligible for this designed study are referred to our eating disorder unit. The quality analysis of FBT will be carried out in all three groups in weekly surveys using the Berner Patienten- und Therapeutenstundenbogen (BPT, English translation, The Bern Post Session Report) [22], an instrument developed in Switzerland to measure the quality of psychotherapeutic treatments. The questionnaires, independently completed by the patients and the therapists, will be matched after each session for the purpose of process analysis. The acceptability in terms of treatment satisfaction will be assessed by the FBB‐HT, which is an adapted version of the Fragebogen zur Beurteilung der Behandlung (FBB, English translation, The Questionnaire of Treatment Assessment) [27]. The FBB is rated on a five‐point Likert scale and administered at the 3-month follow-up assessment.

#### Effectiveness

Adolescents’ heights and weights are to be measured to determine BMI at all follow-up timepoints along with an assessment of eating disorder symptoms using the EDE and the Eating Disorder Inventory (EDI-2; [34]). Both instruments have excellent reliability and validity [7, 12]. The primary outcomes for adolescents are improvement in BMI *z*-score based on the CDC growth charts, as well as a decrease in eating-related psychopathology. Secondary endpoints include stabilization of somatic health status, as measured by vital signs (blood pressure and pulse), improvement in psychosocial well-being and reduction in perceived stress. Accordingly, parents will complete the Perceived Stress Scale (PSS; [5]), which is commonly used to quantify subjective feelings of stress. Adolescents will also complete the Brief Dyadic Scale of Expressed Emotion (BDSEE; German translation by [32]), which captures expressed emotions from the patient’s perspective.

#### Interfamily relations as mediator/moderator

In order to examine how perceived intrafamily stress affects different treatment schemes, at the beginning of treatment and at follow-up assessments, both adolescents and parents are to be interviewed using the short version of the Family Questionnaire (FB-K; German translation, Familienbogen–Kurzversion; [4], a self-report instrument used to assess family function in relation to two aspects: (1) emotional connection between family members and (2) willingness to communicate.

### Power analysis

The target sample size for the primary analysis was derived from an a priori power calculation using G*Power (version 3.1.9.7) and repeated measures ANOVA as a statistical model. Assuming a small effect size of *f* = 0.2, power of 80%, type 1 error level of *p* < 0.05 and correlation among repeated measures of *r* = 0.5, the necessary total sample size of 66 is adequate to test our main hypothesis involving three groups, two time points and the statistical interaction term. Assuming a reasonable attrition rate of 25%, we set our target sample to 90, equivalent to 30 participants per group, assuming equal treatment arm sizes. This number also aligns with the study feasibility, as it approximates the number of eligible patients who previously registered at the outpatient clinic over time intervals similar to the planned recruitment period of 3 years.

### Data analysis plan

The initial descriptive statistics will summarize participant data characteristics as means, standard deviations, minima, maxima, frequencies and percentages. The validity of stratified randomization will be evaluated by inspecting between-group differences at baseline by means of a one-way analysis of variance (ANOVA), Kruskall–Wallis test or chi-squared test (in dependency of conventional statistical assumptions and the variable type, i.e., continuous versus categorical). Outcome data will be evaluated for the normality assumption by a combination of visual (Q–Q plots, histograms) and statistical (Shapiro–Wilk test) methods and will be transformed if necessary to meet the normality assumption. Participant characteristics of the analyzed sample and dropouts will be subjected to an additional inspection for possible non-random differences. Missing data will be handled by the maximum likelihood estimation method. The effects of the interventions will be assessed as changes in the primary outcomes from pre-test to post-test and follow-up assessment using EDE, EDI-2 and BMI analyzed with a repeated measures ANOVA followed by a post-hoc Tukey’s test to compare treatment means when there is a significant effect. The potential influence of confounding factors (age, premorbid BMI, duration of illness, age at onset, duration and setting of pre-treatment, medication use) will be further explored in sensitivity analyses. The secondary continuous variables will be analyzed in parallel. The measures related to treatment implementation (FBB, BPT) will be evaluated using descriptive statistics. Corrected effect sizes, expressed as Hedges’s *g* and significant deviations beyond the 95% confidence interval, will be computed for between- and within-group terms and will indicate empirical power estimates for a subsequent confirmatory study. Exploratory analyses will test the FB-K measure as a mediator/moderator of efficacy. To examine the FBT process, the ratings of each therapy session by the adolescents and the therapists will be compared and analyzed to assess their common development over time. More advanced statistical methods, such as generalized estimating equations (GEE) (which allow for more flexible modelling of the correlation structure among observations, are less sensitive to violation of assumptions and estimate population-wide average effects) will be also applied to assess differences in BMI percentiles and EDE/EDI-2 trajectories. The statistical significance threshold will be set to *p* < 0.05.

## Conclusion

This study protocol builds on the hypothesis of FBT-HT functioning as a well-accepted and effective supplement to FBT [9, 17, 30]. By comparing FBT-HT to both standard outpatient FBT and FBT-MBSR, we can examine the extent to which the different treatment approaches differentially affect treatment outcomes as measured by (1) a change in BMI, (2) eating disorder psychopathology and (3) interfamilial stress experience and communication. We will also explore how different treatment arms are influenced by intrafamily relationships integrated as a mediator/moderator variable. Process analysis can be used to demonstrate how patients as well as therapists perceive (1) the therapy relationship, (2) therapy goal achievement and (3) therapeutic effect factors among the treatment arms. Accordingly, valuable insights with regard to the setting, frequency and therapy duration may be gained. As the responsibility for the therapy process is to be shared and there is to be ongoing exchange between the two therapists involved, we anticipate that the perceived burden on therapists will be lower in the combined groups (FBT-HT; FBT-MBSR). Further data analyses may also involve examining the influence of variables, such as clinical features (e.g., low body weight, high risk for inpatient referral), parental situation, number of siblings and occupation of parents, on the outcome of the different treatment modalities. As an additional consideration, one could also assess the indication for the corresponding treatment through baseline profiling [31]. Future studies will be relevant for more individualized treatment plans to optimize treatment outcomes. Finally, the proposed project is closely aligned with public health priorities to optimize the outcomes of evidence-based treatments and test their effectiveness in community-based settings, while collecting important contextual information to support implementation efforts.

## Data Availability

Data sharing is not relevant to this article because no datasets have yet been created or analyzed as part of this study.
